# Perceived work-ability and its associated factors among nurses working in the Northwest of Amhara regional state Referral Hospitals, Northwest Ethiopia, 2022

**DOI:** 10.1186/s12889-023-16386-0

**Published:** 2023-07-31

**Authors:** Gebremeskel Kibret Abebe, Kibret Asimare Melkie, Addis Wondmagegn Alamaw, Alemu Birara Zemariam, Belayneh Shetie Workneh

**Affiliations:** 1 Department of Emergency and Critical Care Nursing, School of Nursing, College of Medicine and Health Sciences, Woldia University, Woldia, Ethiopia; 2 Department of Surgical Nursing, School of Nursing, College of Medicine and Health Sciences, University of Gondar, Gondar, Ethiopia; 3 Department of Pediatric and Child Health Nursing, School of Nursing, College of Medicine and Health Sciences, Woldia University, Woldia, Ethiopia; 4 Department of Emergency and Critical Care Nursing, School of Nursing, College of Medicine and Health Sciences, University of Gondar, Gondar, Ethiopia

**Keywords:** Work ability, Work ability index, Nurse, Ethiopia

## Abstract

**Background:**

Nurses with reduced work ability had a high risk of disability pension, sickness absences, retirement intention, and leave their job and profession early. Nurses frequently suffer from job related stress, occupational fatigue and sleep problems, which can further compromise their work ability.

**Aims of the study:**

The aim of this study was to assess perceived work ability and its associated factors among nurses working **in the Northwest of Amhara regional state Referral Hospitals, Northwest Ethiopia, 2022.**

**Methods:**

A multicenter, an institutional based, cross-sectional study was conducted among 410 nurses working in five selected Referral hospitals, found **in the Northwest of Amhara regional state, Northwest Ethiopia, 2022**. The data were collected using a structured, self-administered questionnaire and entered using Epi info version 7.2.5 software, analyzed using SPSS version 25. Summary statistics (median or IQR for continuous data and frequency and percentage for categorical variables) were used. The ordinal logistic regression was used to assess’ the presence of association between dependent and independent variables.

**Results:**

The findings of this study revealed that 59.0% of nurses had poor level of work ability, whereas 34.4% and 6.6% of nurses had sub-optimal and optimal level of work ability respectively. Multivariable ordinal logistic regression revealed that being male [AOR = 2.43; 95% CI (1.52, 3.91)], being BSC nurse [AOR = 0.21; 95% CI (0.08, 0.51)], nurses who had poor sleep quality [AOR = 0.34; 95% CI (0.12, 0.98)] and nurses who had chronic disease [AOR = 0.18; 95% CI (0.08, 0.41)] were significantly associated with nurses’ level of work ability, p-value < 0.05.

**Conclusions:**

In this study, the prevalence of poor level of work ability among nurses was high. Nurses with a female sex, nurses who had chronic disease, BSC holders and nurses who had poor sleep quality had a poor level of work ability. The federal Minister of health and the study hospitals collaborative with concerned stakeholders to design strategies to enhance work ability among nurses.

## Background

Work ability is defined as the physical, mental, and functional capacity of workers in their current jobs, considering opportunities, challenges, and demands of the job with available resources [1]. It is a balance between an individual life and work life. The balance includes maintaining health, work ability, occupational well-being as well as coping at work [1]. Work ability is an essential social issue, because it affects the health and well-being of individuals [2].

An inadequate level of work ability has a higher risk of long-term sickness absences from jobs [3], high risk of disability pension, increased premature death. Moreover, employees level of work ability used as a prognostic tool to assess disability [4–6]. Work ability of workers has an impact on retirement intention, leads to getting less income, fewer employment benefits, and causes workers to leave their job and profession early [7–9].

An employee’s level of work ability predicts disability and mortality in life. The rate of death increased with a decreased level of work ability [10]. Furthermore, work ability was a major determinant of quality of life [11, 12]. An inadequate level of work ability hurts employees’ quality of life [8]. Nurses with a poor level of work ability are exposed to such negative impacts.

Among four hospital nurse staff, one nurse member is estimated to have an inadequate level of work ability worldwide [8]. The studies conducted in Europe, revealed that work ability was significantly lower among nurse professionals [13]. Whereas, in Africa, evidence from Egypt and Nigeria showed that 14.1% and 29.2% of health care providers and nurses had inadequate levels of work ability respectively [14, 15].

The nurse is the largest group of employees in any healthcare facility and performs a crucial function in any health care institution. The nurse is unique in patient care and hospital operations [16, 17]. Also, a nurse is a means of communication between physicians and patients [18]. The nurse faces a high risk for occupational fatigue, work-related occupational stress, and sleep problems [19–22], those factors affected the nurse’s level of work ability [23–25].

Even though several studies were conducted on workability among nurses and health care workers in the developed world, to the best of the authors’ knowledge, there is limited evidence on nurse’s level of work ability in resource-limited countries, including Africa, especially in Ethiopia. In Ethiopia, hospital working conditions, work load, working hours and environmental and administrative factors are quite different from the developed world. Also, in Ethiopia there are disproportionate between patient nurse ratio, and absences of shift work among nurse may affect nurses’ workability. To this fact, it valuable to assess perceived workability and its associated factors of workability among nurses working in referral hospitals found at Northwest of Amhara regional state, Northwest Ethiopia.

## Significances of the study

The finding of this study primarily used for nurses to enhances their level of work ability by promoting health, and decreasing factors hindering workability. Also, the finding used for health care programmers and stakeholders are help to implement and set up strategies to promote nurses’ level of work ability in Hospital episode of care. Moreover, these studies are used as a baseline data for a researcher to conduct studies nationwide among nurses, or all health care workers and even help to study for other employees other than health care workers. Also, the findings of this study were not limited to Ethiopia, but the findings may be used as preliminary information for future researchers ‘plan to conduct research in other African countries.

## Methods

### Study design and settings

The study was conducted from June 1, 2022 to June 30, 2022, using an institutional-based, cross-sectional study design. The study was conducted in referral hospitals found in the Northwest part of the Amhara regional state, Northwest Ethiopia. Debre Markos, Tibebe Ghion, Debre-tabour, Felge-hiwot and the University of Gondar are the five referral hospitals found in the Northwest part of Amhara Regional State. Each referral hospital serves 3.5–5 million people [26]. Each referral hospitals had emergency, Intensive care unit, recovery, Operating room, inpatient, medical, surgical and outpatient departments. According to data obtained from each hospital director, there were a total of 1756 nurses working in the five referral hospitals [27]. Among which 161 nurses, 679, 416, 292, and 208 of nurses working at Debre-tabour, University of Gondar, Felge-hiwot, Tibeb-gioun and Debre-markoes referral hospitals respectively.

### Sources and study population

#### Sources population

The sources populations for this study was all nurses who are working in in the Northwest of Amhara regional state Referral Hospitals, Northwest Ethiopia.

#### Study population

All nurses working in the Northwest of Amhara regional state Referral Hospitals, Northwest Ethiopia and present during study period was a study population.

### Inclusion and exclusion criteria

All nurses who are qualified as diploma holders and above and working at selected Referal hospitals and who are present during data collection were included in the study. Nurses who had less than six months of total work experience were excluded.

### Sampling

The sample size was calculated by using a single population proportion formula by taking the estimated proportion of inadequate workability at 50%, a confidence level of 95%, and a margin of error of 5%. The final sample size was 422, after adding a 10% non-response rate. The study participants from the five hospitals were selected using a proportional allocation technique. From Debre-tabor referral hospital out of 161 nurses (n = 39), University of Gondar referral hospital of 679 nurse (n = 163), Felge-hiwot referral hospital of 416 nurses (n = 100), Tibebe Ghion referral hospital of 292 nurse (n = 70), Debre Markos referral hospital out of 208 nurse (n = 50) were allocated. Moreover, to select the actual sampled nurses in each hospital, a simple random sampling technique was applied using a lottery method. To begin with, the sampling frame was prepared by taking the lists of nurses from the hospital’s human resource management.

### Operational definitions

Nurse: - is a professional who is registered with the Ethiopian ministry of health and has a diploma and above.

The overall score on the self-reported Work Ability Index measurement ranged from 7 to 49. A score of < 37 indicates poor work ability, a score of 37–43 indicates suboptimal, and a score of 44–49 indicates optimal level of work ability [14, 28].

Sleep quality is measured based on a single item sleep quality scale (SQS) on a visual scale of 0–10. A score of 1–3 shows poor sleep quality, a score of 4–6 shows fair sleep quality, a score of 7–9 shows good sleep quality, while a score of 10 shows an excellent sleep quality [29].

Physical activity was classified as moderate, low and intense using Guidelines for Americans. To classify nurses who had moderate physical activity, the nurses may respond yes to one of the questions prepared to assess’ moderate physical activity [30].

Also, nurses’ physical activity is classified as intense physical activity when they respond yes to one of the questions prepared to assess’ intense physical activity [30].

Whereas, nurses’ physical activity is categorized as low when they respond yes to one of the questions to assess low physical activity [30].

Body mass index of participants were categorized based on WHO classification as < 18.5kgm^2^ (underweight), 18.5kgm2 -24.9kgm^2^ (normal body weight), 25.0kgm^2^- 29.9kgm^2^ (over weight) and > 30.0kgm^2^ (obesity) [31].

### Study variables

#### Dependent variables

The dependent variable for this study was the level of work ability among nurses. This includes poor, sub-optimal and optimal level of work ability.

#### Independent variables

**Sociodemographic variables**: Age, sex, job experience, marital status, educational status.

Personal-related variables: Body mass index, physical exercise activity, the presence of chronic disease, presence of cardio vascular disease, musculoskeletal disease, gastro intestinal disease, respiratory disease, renal disease, liver disease, mental disease.

Work-related variables: job related stress, sleep quality, work-related fatigue, working hours, working more than current hospital places/24 hours, current working unit.

### Data collection instruments and procedures

Quantitative data was collected using a structured pre-tested self-administered questionnaire. The questionnaire had four sections. The first section contains five questions regarding the socio-demographic characteristics of the participants, the second section contains ten questions regarding personal related characteristics of participant’s conditions as nurses, and the third section contains four work related questions. From a work related questionnaire, the sleep quality of participants was measured with the newly developed single-item question, which is a validated and reliable scale to measure sleep quality. The scale measures sleep quality based on sleep on 7 days and ranges from 0 to 10 with visual scale measurements. A score of 1–3 indicates poor sleep quality, a score of 4–6 indicates fair sleep quality, a score of 7–9 indicates good sleep quality and a score of 10 shows excellent sleep quality [29] were adopted from the previous study. The WAI questionnaire is an English version of questionnaire complied by study participants. WAI validating and reliable questionnaire confirmed by previous studies [32]. It consists of 7 dimensions, such as 1, Current work ability compared to lifetime best(score 0–10) 2, subjective current work ability regarding physical and mental demand (two question 5 point scale) 3, the number of diseased diagnosed by a physician (1 = > 5 to 7 = 0) 4, estimated work impairment due to disease (sick point sale 1–6) 5, sick leave in the last 12 months(score range 5 = 0) 6, person progress of work ability in the next two years (score 1 hard,4 not sure) 7, mental resources in the past few months asses three dimensions daily activity, being active and feeling hope to the future (never 0, always 4). The total score ranges from 7 to 49. A score of 7–27 points corresponds to low work ability,28–36 points correspond to moderate work ability,37–43 corresponds to good work ability and 44–49 score corresponds to excellent work ability [28]. In this study, we used three sub-scales where a score < 37 corresponds to poor work ability, a score of 37–43 corresponds to sub-optimal work ability and a score of 44–49 corresponds to optimal work ability [14, 28].

Physical activity was assessed using guidelines for Americans. Physical activity is classified as moderate, low and intense. Moderate physical activities include walking energetically (2.5 miles per hour or faster), Leisure swimming, bicycling slower than 10 miles per hour on level terrain, tennis, active forms of yoga, ballroom or line dancing, general hard work and home repair work exercise classes like water aerobics [30].

Intense physical activity includes jogging or running, swimming laps, tennis (singles), vigorous dancing, bicycling faster than 10 miles per hour, jumping rope, heavy yard work (digging with heart rate increases), hiking uphill or with a heavy backpack, high-intensity interval training, exercise classes like vigorous step aerobics or kickboxing [30].

Low physical activity, includes light walking, stretching, lifting hand weight, pushups against walls [30]. The data collector was administering a questionnaire to study participants and instructed the participants to read the instructions and fill in their answers carefully. During data collection, data collectors and supervisors followed the recommended precautions to prevent COVID-19.

### Statistical analysis

The data were entered using Epi info version 7.2.5 software, then, exported, checked, sorted, categorized, coded and analyzed using SPSS version 25. Initially, the data were checked for missing data and outliers. There were no missing data. The study population were described in terms of the relevant variables using summary statistics (median or IQR for continuous variables and percentage and frequency for categorical variables). The data were presented using texts, tables and figures. Bivariable analysis was used to see the association between each determinant and the outcome variable by using ordinal logistic regression. The assumption of a proportional model was met. The model fitness was tested using deviances - the 2 log likelihood ratio reveals p-value = 0.000 and the goodness of fit was tested by Pearson and, deviances chi-square, which reveals p-value= (0.449 and 1.000 respectively), while the parallel line test result revealed p-value = 0.138. All variables with P < 0.25 in the Bivariable analysis were included in the final model of multivariable analysis in order to control all possible confounders. The correlation between independent variables was tested for multi co-linearity by using the variance inflation factor (VIF = 3.643), which is thought to be suggestive of no multi co-linearity. The direction and strength of the statistical association was measured with an adjusted odds ratio along with a 95% CI estimated and a P-value < 0.05 was considered statistically significant.

### Data quality management

The questionnaire was prepared in English and then translated into Amharic language and then back to English by a language expert person to check its consistency and contextualization. Pre-testing was done on 5% [22] of the total study-appropriate participants at Woldia Hospital, who weren’t from the hospital under study but had the same health facility characteristics. This was done prior to the real data collection. In the present study, the scale’s face validity was examined by research experts. Cronbach alpha was tested, and the results showed that WAI 0.78. The principal investigator was provided one-day training for data collectors and supervisors on the objectives, tools, and process of data collection, as well as how to maintain confidentiality of information provided by study subjects. Three diploma nurses were given the task of collecting the data, and a BSc nurse was given the task of supervision of the entire data collection. During the data collection period, the supervisor checked whether the questionnaire was correctly filled or not and also made close supervision of data collectors and made spot-check and reviewed the data collection process completed daily to ensure completeness and consistency of the information that was collected. Furthermore, data is also checked during entry onto the computer before analysis.

## Results

### Socio-demographic characteristics of respondents

A self-administered questionnaire was distributed to 422 nurses. Out of these, 410 completed the questionnaire, which yielded a response rate of 97.2%. The respondents’ age ranged from 22 to 56; the median age was 30.00 years (inter-quartile range: 5 years). The majority of study participants, 259 (63.17%), were between the ages of 21 and 30. 246 (60%) of the respondents were men. Regarding marital status, 233 (56.83%) of the respondents were married (Table 1).

**Table 1:** Distribution of Socio-demographic related characteristics for the study perceived work-ability and its associated factors among nurses working in the Northwest of Amhara regional state Referral Hospitals, Northwest Ethiopia, 2022, (n = 410)

Variables	Category	level of work ability			Total
		Poor	Sub-optimal	Optimal	
Age	21–30 year	147(56.8%)	94(36.3%)	18(6.9%)	259(63.17%)
	30–40 year	88(65.7%)	38(28.4%)	8(6.0%)	134(32.6%)
	> 40 year	7(41.2%)	9(52.9%)	1(5.9%)	17(4.23%)
	Total	242(59.0%)	141(34.4%)	27(6.6%)	410(100%)
Sex	Male	127(51.6%)	93(37.8%)	26(10.6%)	246(60%)
	Female	115(70.1%)	48(29.3%)	1(0.6%)	164(40%)
	Total	242(59.0%)	141(34.4%)	27(6.6%)	410(100%)
Martial statues	Unmarried****	99(55.9%)	65(36.7%)	13(7.3%)	177(43.17%)
	Married	143(61.4%)	76(32.6%)	14(6.0%)	233(56.83%)
	Total	242(59.0%)	141(34.4%)	27(6.6%)	410(100%)
Educational status	Diploma	7(50.0%)	6(42.9%)	1(7.1%)	14(3.41%)
	BSC	227(61.0%)	125(33.6%)	20(5.4%)	372(90.73%)
	MSC	8(33.3%)	10(41.7%)	6(25.0%)	24(5.86%)
	Total	242(59.0%	141(34.4%)	27(6.6%0	410(100%)
Total-work-experiences	< 5 year	103(55.4%)	74(39.8%)	9(4.8%)	186(45.36%)
	5–10 year	103(60.2%)	54(31.6%)	14(8.2%)	171(41.70%)
	> 10 year	36(67.9%)	13(24.5%)	4(7.5%)	53(12.94%)
	Total	242(59.0%)	141(34.4%)	27(6.6%)	410(100%)

### Personal and individual characteristics of respondents

Of the 410 study respondents, 338 (82.44%) had a normal body mass index, which ranges from 18.5 kg/m2 to 24.9 kg/m2, and 28 (6.85%) were overweight. Concerning physical activity, 233 (56.84%) respondents reported low physical activity. Among the study respondents, 53 (12.9%) of the participants had reported the presence of a chronic disease diagnosis by a physician. 20.75%, and 16.98% of respondents had reported musculoskeletal and cardio-vascular disorders respectively, while 16.98%, 9.43%, 16.98%, 3.66% and 1.88% of respondents reported respiratory, mental, gastrointestinal, liver and renal disorder respectively. The remaining respondents had other types of chronic disease (Table 2).

**Table 2:** Distribution of personal-related characteristics for the study perceived work-ability and its associated factors among nurses working in the Northwest of Amhara regional state Referral Hospitals, Northwest Ethiopia, 2022, (n = 410)

Variables	Category	Work ability			Total
		Poor	Sub-optimal	Optimal	
BMI	< 18.5 kg/m2	20 (45.5%)	18 (40.9%)	6 (13.6%)	44 (10.73%)
	18.5-24.9 kg/m2	202 (59.8%)	116 (34.3%)	20 (5.9%)	338 (82.44%)
	≥ 25.0 kg/m2	20 (71.4%)	7 (25.0%)	1 (3.6%)	28 (6.83%)
	Total	242 (59.0%)	141 (34.4%)	27 (6.6%)	410 (100%)
Physical exercise activity	Intense	16 (59.3%)	8 (29.6%)	3 (11.1%)	27 (6.58%)
	Moderate	79 (52.7%)	59 (39.3%)	12 (8.0%)	150 (36.58%)
	Low	147 (63.1%)	74 (31.8%)	12 (5.2%)	233 (56.84%)
	Total	242 (59.0%	141 (34.4%)	27 (6.6%)	410 (100%)
Chronic disease	Yes	45 (84.9%)	7 (13.2%)	1 (1.9%)	53 (12.9%)
	No	197 (55.2%)	134 (37.5%)	26 (7.3%)	357 (87.1%)
	Total	242 (59.0%)	141 (34.4%)	27 (6.6%)	410 (100%)

### Work-related characteristics of respondents

A majority of study participants 319 (77.8%) were worked only at one hospital. On the other hand, 22.2% of respondents were worked at another health facility in addition to their hospital. Nearly half (54.14%) of respondent’s had worked ≤ 8 h per day. Regarding respondents working unit, 27.2%, 22.2%, and 15.85% were worked at surgical ward, medical ward and ICU respectively. 8% of respondents reported poor sleep quality (Table 3).

**Table 3:** Distribution of work-related characteristics for the study perceived work-ability and its associated factors among nurses working in the Northwest of Amhara regional state Referral Hospitals, Northwest Ethiopia, 2022, (n = 410)

Variables	Category	level of work ability			Total
		Poor	Sub-optimal	Optimal	
working other than this hospital	No	189(59.2%)	112(35.1%)	18(5.6%)	319(77.8%)
	Yes	53(58.2%)	29(31.9%)	9(9.9%)	91(22.2%)
	Total	242(59.0%)	141(34.4%)	27(6.6%)	410(100%)
working hours/24 hour	≤ 8 h	133(59.9%)	75(33.8%)	14(6.3%)	222(54.14%)
	> 8 h	109(58.0%)	66(35.1%)	13(6.9%)	188(45.86%)
	Total	242(59.0%)	141(34.4%)	27(6.6%)	410(100%)
current working department	medical ward	53(58.2%)	31(34.1%)	7(7.7%)	91(22.2%)
	surgical ward	72(64.3%)	33(29.5%)	7(6.3%)	112(27.32%)
	ICU	36(55.4%)	27(41.5%)	2(3.1%)	65(15.85%)
	OPD	25(59.5%)	16(38.1%)	1(2.4%)	42(10.24%)
	ED	24(47.1%)	19(37.3%)	8(15.7%)	51(12.43%)
	pediatric ward	32(65.3%)	15(30.6%)	2(4.1%)	49(11.95%)
	Total	242(59.0%)	141(34.4%)	27(6.6%)	410(100%)
Sleep quality	Poor	21(63.6%)	11(33.3%)	1(3.0%)	33(8.0%)
	fair	125(65.1%)	56(29.2%)	11(5.7%)	192(46.8%)
	good	84(54.2%)	60(38.7%)	11(7.1%)	155(37.8%)
	Excellent	12(40.0%)	14(46.7%)	4(13.3%)	30(7.4%)
	Total	242(59.0%)	141(34.4%)	27(6.6%)	410(100%)

ICU -Intensive care unit, ED-emergency department, OPD- out patient department

### Perceived work ability among nurses

The level of work ability was computed from the work ability index total score. The WAI score of respondents ranged from 22 to 45. The median value was 36.00 (IQR = 9). From the total of 410 respondents 242 (59.0%), CI= (53.7, 64.3) had poor level of work ability, while 34.4% CI= (29.5, 39.5) and 6.6%, CI= (4.1, 9.0) had sub-optimal and optimal level of work ability respectively.

### Factors associated with perceived work ability

First, a bi-variable analysis was performed to identify the determinants of levels of work ability. Accordingly, sex, sleep quality, presence of chronic disease, current working department, physical exercises, total work experiences, educational status and work-related fatigue (acute work-related fatigue, chronic work-related fatigue and recovery from work-related fatigue), were eligible for the multi-variable analysis (p < 0.25). In the multi-variable model, sex, presence of chronic disease, sleep quality and educational status, were significant predictors of level of work ability among nurses working at Referral hospitals (p < 0.05).

Nurses of the male sex had 2.43 times [AOR = 2.43; 95% CI (1.52, 3.91)] higher odds of a higher level of work ability compared to being female nurses, given that other variables in the model held constant. The odds of having a higher level of work ability being nurses who had chronic disease were decreased by 82% [AOR = 0.18; 95% CI (0.08, 0.41)] compared to those being nurses who had no chronic disease. The odds of having a higher level of work ability being nurses who had poor sleep quality were decreased by 66% [AOR = 0.34; 95% CI (0.12, 0.98)] compared to those being nurses who had excellent sleep quality. The odds of having a higher level of work ability being a BSC nurse were decreased by 79% [AOR = 0.21; 95% CI (0.08, 0.51)] than nurses of MSC holder (Table 4).

**Table 4:** Results of bi-variable and multi-variable ordinary logistic regression of factors associated with perceived work-ability among nurses working in the Northwest of Amhara regional state Referral Hospitals, Northwest Ethiopia, 2022, (n = 410)

Variables	Category	Work ability			COR with 95% CI	AOR with 95% CI	p- value
		Poor n = 242	Sub-optimal n = 141	Optimal n = 27			
Sex	Male	127	93	26	2.40(1.59,3.64)	2.43(1.52,3.91)	< 0.001**
	Female	115	48	1	1	1	
Educational status	Diploma	7	6	1	0.39(0.11,1.38)	0.27(0.06,1.13)	0.073
	BSC	227	125	20	0.25(0.11,0.56)	0.21(0.08,0.51)	0.001**
	MSC	8	10	6	1	1	
Physical exercise	Intense	16	8	3	1.28(0.58,2.82)	2.27(0.94,5.48)	0.069
	Moderate	79	59	12	1.54(1.02,2.31)	1.40(0.89,2.20)	0.148
	Low	147	74	12	1	1	
Chronic disease	Yes	45	7	1	0.22(0.10,0.48)	0.18(0.08,0.41)	< 0.001**
	No	197	134	26	1	1	
Work experience	< 5 years	103	74	9	1.56(0.83,2.94)	1.81(0.84,3.89)	0.129
	5–10 years	103	54	14	1.38(0.73,2.62)	1.22(0.58,2.57)	0.598
	> 10 year	36	13	4	1	1	
Sleep quality	Poor	21	11	1	0.36(0.14,0.97)	0.34(0.12,0.98)	0.046*
	Fair	125	56	11	0.36(0.17,0.75)	0.48(0.21,1.08)	0.075
	Good	84	60	11	0.55(0.261,1.17)	0.73(0.33,1.66)	0.457
	Excellent	12	14	4	1	1	
Current working unit	Medical ward	53	31	7	1.39(0.68,2.84)	1.04(0.47,2.30)	0.932
	Surgical ward	72	33	7	1.08(0.54,2,16)	1.02(0.47,2.19)	0.968
	ICU	36	27	2	1.43(0.67,3.05)	1.07(0.47,2.46)	0.869
	OPD	25	16	1	1.22(0.52,2.84)	1.27(0.51,3.19)	0.612
	ED	24	19	8	2.41(1.10,5.29)	1.94(0.81,4.65)	0.138
	Pediatric ward	32	15	2	1	1	

*Statistically significant at p-value < 0.05, ** highly statistically significant < 0.001, references category is optimal level of work ability, AOR: Adjusted odds ratio; CI: Confidence interval, COR: crude odds ratio: ED-emergency department,

## Discussion

The aim of this study was to determine perceived work ability and its associated factors among nurses working in the Northwest of Amhara regional state Referral Hospitals, Northwest Ethiopia. The result of the current study showed that 242(59.0%) (CI = 53.7%, 64.3%) nurses had poor level of work ability, which is higher than the studies done in Egypt [14] and Nigeria [15]. In Nigeria, 29.2% of study participants had a poor level of work ability [15]. The possible justification for discrepancies could be due to varies in inclusion and exclusion criteria. The study in Nigeria excludes nurses who had previous accidents or injuries and includes part-time workers. This leads to an overestimated level of work ability [15]. The tool of work ability Index is the aggregated sum of chronic disease, which includes accident or injury [15], which might be the cause for discrepancy.

In this study, the prevalence of poor level of work ability was higher than the study done in Egypt (14.1%) [14]. The possible explanation for these differences might be methodological differences, socioeconomic development differences, organizational and work-related factors. In Egypt, the study subjects were all health care providers [14], whereas, in this study, the study subjects were nurses. Compared to other health care professionals, nurses are more likely to suffer from work-related fatigue, job stress and sleep problems [19–22], which in turn adversely affect degree of work ability [23–25].

In this study, the prevalence of poor level of work ability was higher than the studies done in Brazil (43.3%) [33], Australia (8.5%) [34], and Iran (34.7%), (33%) [25, 35]. The difference in the study setting, study population, organizational structure, and socio-economic status might be the causes of the discrepancy. In Brazil, a single hospital was used for the study, which may not be sufficiently representative [33]. Additionally, in Brazil, the organizational structure and administrative of the hospital may be favorable for nurses, which increased their level of work ability. In fact, the work ability of employees is influenced by demands of jobs, work communities, organizations, and overall work cultures [1].

In Iran, the study’s participants included medical professionals as well as other employees from the banking and manufacturing industries [25]. Since these groups of employees’ work environments differed in terms of their physical and mental needs, which results in unequal measurements of work ability. Other reasons for this variation might be that healthcare providers are exposed to high levels of physical, mental and emotional stressors that can influence their performance and efficiency and have a negative impact on their work ability [36].

The finding of this study revealed that the prevalence of poor level of work ability was higher than the study done in Bangladesh (7.1%) [37]. The possible explanations for these discrepancies could be methodological differences, like sample size and study area. In Bangladesh, the sample size was 197, and the study was conducted only in one hospital, which might have an impact on the prevalence of work ability and reduced the study’s representativeness.

Likewise, the prevalence of poor level of work ability in the current study was 59.0%. This finding was comparable with the study done in Iran (57%) [23]. This similarity might be due to similarity in study design, study subjects and work-related factors. The study in Iran was conducted on nurses working in an emergency department were more stressful than nurses working on another ward [23]. Also in this current study, the study subjects were nurses, who are more vulnerable to job related stress, sleep problem and work-related fatigue [19–22]. In fact, those factors had negative impacts on level of work ability.

The current study showed that sex, the existence of chronic diseases, sleep problems, and educational states were the main predictors of work ability among nurses working at comprehensive specialized hospitals. Sex was one of the predictors of work ability in this study. Being male nurses had 2.43 times higher odds of a higher level of workability compared to being female nurses. This finding was consistent with the study conducted in Egypt [14], Brazil [38] and Australia [34]. The possible reason might be female nurse had long hours of domestic work and workload [39]. In Ethiopia, female nurses had multiple roles outside the workplace, such as care of their children. On another studies conducted in Slovakia, Brazil and Iran [40–42] showed that gender disparity had no effect on level of work ability. These differences could be due to methodological variation and national socio-cultural differences.

The current study showed educational status was a significant predictor of nurse work ability. The odds of having a higher level of work ability among BSC nurses decreased by 79% compared to those nurses of MSC holders. This finding was supported by a study conducted in Poland, Croatia and Bangladesh [37, 43, 44]. The possible justification might be that people with lower levels of educational status commonly take on work involving more physical labor than those with higher education, which might contribute to unhealthy work practices in employees [45]. Additionally, nurses with lower educational status had lower job motivation than those with higher educational status [46], which in turn leads to a low level of work ability [45].

In this study, the existence of chronic disease were the significant a predictor of a nurse’s level of work ability. The odds of having a higher level of work ability being nurses who had chronic disease were decreased by 82% compared to those nurses who had no chronic disease. This finding was supported by studies in Egypt [14] and Japan [41]. If a respondent had a chronic illness, their level of work ability was reduced because, among the seven dimensions of the work ability index tool, some of the tool value depends on the presence of chronic illness and sick leave. Additionally, the presence of chronic illnesses including cardiovascular, musculoskeletal, and respiratory conditions causes sick days and impacts frequent engagement in physically and mentally demanding jobs, which results in reduced workability [47, 48]. Deteriorating health had a negative impact on work ability [1].

The present study showed that sleep problems were associated with level of work ability. The odds of having a higher level of work ability among nurses who had poor sleep quality were decreased by 66% compared to those nurses who had excellent sleep quality. This finding was supported by a study conducted in Iran [25]. The possible explanation might be sleep problem affecting health [49], poor sleep quality had strong associations with physical and mental health and sickness absences [50], this finding in turn affect level of work ability.

Implications of the present studies for health care programmers and stakeholders are help to implement and set up strategies to promote nurses’ level of work ability, such as, decrease workload, nutrition programs, exercise activists on hospital episode of care. Moreover, these studies are used as a baseline data for a researcher to conduct studies nationwide among nurses, or all health care workers and even help to study for other employees other than health care workers. Also, the findings of this study were not limited to Ethiopia, but the findings may be used as preliminary information for future researchers ‘plan to conduct research in other African countries.

## Conclusion

In this study, the prevalence of poor level of work ability among nurses was high. Being a female nurse, having chronic disease, poor sleep quality and BSC nurses’ holders negatively affect nurses’ level of work ability. This suggests that initiatives to enhance a nurse’s level of work ability should be taken into account. The Ethiopian Federal Minister of Health should develop strategies to enhance nurses’ level of work ability in the institutions under study. A nation-wide study should be needed regarding sources of low level of work ability and strategies to enhance the level of work ability in the Ethiopian hospital setup.

### Strength and limitation of study

This research is the first research conducted in Ethiopia regarding work ability and its associated factors among nurses, which was used as baseline information for different stack-holders and researchers. The study was conducted on both nurses including nurses who had leader role and a matron, there might be underestimation or overestimate of the results. Moreover, further study is required to discover the level of work ability among nurses and nurses who had a leader role.

## Data Availability

All data are available upon request. The reader could contact the corresponding author for the underlying data.

## List of abbreviations

AOR: Adjusted Odds Ratio
BSC: Bachler of Sciences
BMI: Body Mass Index
CI: Confidence Interval
MSC: Master of Science
SQS: Single Item Sleep Quality Scale
SRS: Systematic random sampling
SPSS: Statistical Package for Social Sciences
SQS: Single Item Sleep Quality Scale
WAI: Work Ability Index
WHO: World Health Organization
